# A Longitudinal Study of the Bidirectional Relations Between Anxiety Symptoms and Peer Victimization in Urban Adolescents

**DOI:** 10.1177/0886260518824647

**Published:** 2019-01-18

**Authors:** Tess K. Drazdowski, Wendy L. Kliewer, Albert Farrell, Terri Sullivan, Roxann Roberson-Nay, Lena Jäggi

**Affiliations:** 1 Virginia Commonwealth University, Richmond, USA; 2 UCLA Integrated Substance Abuse Programs, USA; 3 Oregon Social Learning Center, Eugene, USA; 4 University of Basel, Basel, Switzerland

**Keywords:** bullying, mental health and violence, cultural contexts, youth violence

## Abstract

The current study examined bidirectional relations between anxious symptoms and two forms of peer victimization (i.e., overt and relational) within an underrepresented sample of urban adolescents during key transition periods (i.e., elementary to middle school; middle school to high school) and the following 2 years. A predominantly African American sample (91%) of 358 adolescents (56% female, mean age = 12.10 years) living in low-income urban areas were assessed annually across 4 years. Using self-report measures, adolescents reported on their past year experiences of anxiety and peer victimization. Longitudinal path analyses tested progressively complex models for each type of victimization. Anxious symptoms predicted both overt and relational victimization at the time of transition (Wave 1 to Wave 2) and the following year (Wave 2 to Wave 3). Furthermore, whereas previous levels of victimization and future anxious symptoms were positively correlated over time, only relational victimization at Wave 1 predicted anxious symptoms at Wave 2. Prior levels of each construct were the strongest predictor of future outcomes (e.g., anxious symptoms at Wave 1 predicting anxious symptoms at Wave 2). Overall, there was little support for bidirectional relations between anxiety symptoms and peer victimization. Intervention and prevention programs seeking to reduce peer victimization or anxiety should start by targeting the symptom/behavior of interest. Interventions that target anxious thoughts and feelings during these key transition times in adolescence should be assessed as areas of priority.

Peer victimization (PV), defined as being maltreated by one’s peers, including being exposed to such behaviors as hitting, name-calling, and being purposefully excluded from groups, is a common problem among adolescents that has been shown to have serious consequences. Student reports from nationally representative samples indicate that 16% to 20% of youth in the United States experience some form of PV, though these rates vary by age, sex, ethnicity/race, and disability status (Blake, Lund, Zhou, Kwok, & Benz, 2012; Kann et al., 2016). For example, for African American youth, rates range from 13% to 29% in nationally representative samples, while rates range from 24% to 29% for European American youth (Kann et al., 2016). A large literature highlights associations between PV and many negative outcomes such as internalizing and externalizing behaviors (for reviews, see Casper & Card, 2017; Klomek, Sourander, & Gould, 2010; Reijntjes et al., 2011; Reijntjes, Kamphuis, Prinzie, & Telch, 2010). This has raised concern from parents, the school community, and legislators partly as a result of bullying-related suicides and other highly publicized incidents in schools (e.g., Curry, 2012), as well as a documentary *Bully* (Hirsch & Lowen, 2012). These all have highlighted bullying and PV as priority problems for today’s youth. In response to these reports, the United States federal government created a website managed by the U.S. Department of Health & Human Services to help increase the country’s anti-bullying efforts (stopbullying.gov; Calmes, 2011).

Researchers have distinguished between several types of PV, with two key subtypes being overt and relational victimization. Overt victimization involves both openly aggressive nonphysical and physical person-to-person acts such as name-calling or pushing, shoving, or hitting, respectively. Alternatively, relational victimization includes more covert behaviors such as being excluded from a group, deliberately ignored, or the target of rumors or lies that damage the victim’s reputation and social relationships (Crick & Bigbee, 1998). Experiences and frequencies of overt and relational victimization vary according to sex, age, outcomes, and how they are perceived by parents and teachers (Casper & Card, 2017), which provides support for considering these types of victimization as distinct constructs. In addition, researchers investigating the structure of PV, including measurement invariance and developmental change in different types of victimization, found support for a two-factor model consisting of overt and relational victimization items as compared with a one-factor victimization model or a three-factor model consisting of relational, physical, and verbal victimization (Rosen, Beron, & Underwood, 2012). This provides evidence supporting overt and relational victimization as unique constructs. Though researchers have begun to investigate overt and relational victimization and their consequences separately (e.g., Casper & Card, 2017; Ranta, Kaltiala-Heino, Fröjd, & Marttunen, 2012), many researchers still combine overt and relational victimization into a single measure of PV (e.g., Kochel, Ladd, & Rudolph, 2012).

The negative consequences of PV can be severe and can have long-term impacts on victims’ well-being, even into adulthood (Faith, Storch, Roberti, & Ledley, 2008; Meltzer, Vostanis, Ford, Bebbington, & Dennis, 2011). Researchers have established associations between being a victim of PV and increased psychosomatic complaints (Nixon, Linkie, Coleman, & Fitch, 2011) and poorer academic achievement (Nakamoto & Schwartz, 2010) as compared with nonvictims. Victimized youth may also exhibit more frequent externalizing behaviors, such as aggression, delinquency, and drug use (e.g., Reijntjes et al., 2011), internalizing symptoms, such as anxiety and depression (for reviews, see Casper & Card, 2017; Reijntjes et al., 2010), and more posttraumatic stress disorder (PTSD) symptoms (Idsoe, Dyregrov, & Idsoe, 2012) than their nonvictimized peers.

Although researchers have explored relations between victimization and a variety of adjustment problems, there has been increasing focus on its association with internalizing symptoms. Some researchers have theorized that although internalizing symptoms may exist before a youth’s exposure to PV, the frequency of symptoms may escalate as a result of the PV (Bernstein & Watson, 1997). Victimized youth are at an increased risk for suicidal ideation and attempts, particularly if they are experiencing internalizing symptoms (for reviews, see Klomek et al., 2010; Van Geel, Vedder, & Tanilon, 2014). It is also important to focus on internalizing symptoms because these symptoms are easier for both parents and teachers to overlook. Unlike externalizing behaviors, internalizing symptoms occur by their very nature within the child and might thus not always be as easily as observed. Therefore, these youth may be at an increased risk of not receiving the help and support they need to learn better ways to address instances of victimization (Singh & Bussey, 2011).

The majority of research examining relations between PV and internalizing symptoms has focused on depressive symptoms (e.g., Tran, Cole, & Weiss, 2012). A meta-analysis by Hawker and Boulton (2000) found that while depression had the strongest association with PV, there was also a significant association between anxious symptoms and PV. There is some evidence, albeit equivocal, for a bidirectional relation between PV and anxiety symptoms. Studies that have investigated anxiety and PV have primarily focused on social phobia symptoms (Mulder, Hutteman, & Van Aken, 2017; Ranta et al., 2012; Siegel, La Greca, & Harrison, 2009).

Prior research examining the bidirectional relations between PV and anxiety, including internalizing symptoms more broadly, has had a variety of limitations. Contradictory results may be the result of samples drawn from populations that differ based on age and culture (e.g., race/ethnicity, country), or because the length of time between data collections of the studies varies. Researchers also have been inconsistent in their definitions of PV and have not always investigated the unique constructs that comprise these domains (i.e., overt and relational victimization). Furthermore, researchers have tended to combined anxious and depressive symptoms (Eastman et al., 2018; for a review, see Reijntjes et al., 2010).

In addition, the majority of samples examined in these studies have been limited to those in sixth grade or younger, and most consist of primarily Caucasian youth. Also, almost all the research completed so far gathered data at only two time points across a timeframe of 2 years or less. This can be problematic as certain sensitive periods may be missed. For example, naturally occurring transition times, such as the transition from elementary school to middle school, or middle school to high school, may offer youth opportunities to either remove themselves from environments that included PV and establish new healthier peer relationships or enter new environments where PV is more prevalent and/or supportive peer relationships have been discontinued. Furthermore, there has yet to be a study that analyzes anxiety symptoms broadly in relation to overt and relational PV over time.

We designed this study to contribute to the literature on PV by addressing several of the limitations of past work. First, we considered general anxious symptoms as a unique construct, going beyond work that has focused solely on social phobia symptoms. Second, we examined overt and relational victimization separately. Third, we investigated the relation between anxious symptoms and victimization across 3 years using four time points encompassing youth in fifth through 11th grade. This captured a broader range of development than past studies and allowed for a more detailed investigation of the timing of these relations. Fourth, we investigated the potential bidirectional nature of the relations between anxious symptoms and PV with path analyses using cross-lagged path models, which allow for a more comprehensive examination of this phenomenon. Finally, the community-based sample consisted of poor, mostly African American youth who are underrepresented in the literature on PV. This gap in the literature reflects a general trend in psychological research to understudy ethnic minority populations (Sue, 1999). Based on prior research and theory, we hypothesized bidirectional relations between anxious symptoms and PV over time.

## Method

### Participants

The sample consisted of 358 urban youth (*M* age in years = 12.10, *SD* = 1.63; 56% female) from a larger longitudinal study focusing on youth violence exposure, physiology, and drug use (see Kliewer, Dibble, Goodman, & Sullivan, 2012). Youth were in either fifth grade (younger cohort: *n* = 191, 56%) or eighth grade (older cohort: *n* = 167, 44%) at Wave 1 of the study (2005). Most (91%) identified themselves as African American, 3% as European American, 3% as American Indian, and 3% endorsed other racial/ethnic groups. The most common family structure consisted of maternal caregivers who never married (40%), followed by those who were married or cohabitating (32%), separated or divorced (26%), and widowed (2%). The median household income for the sample was between US$300 and US$400 per week, with 34% of the sample earning US$300 or less per week and 29% earning a weekly income of US$500 or more. Caregivers’ level of education varied with 23% who did not complete high school, 31% holding a high school or general education diploma, 24% who pursued but did not complete some form of education beyond high school, 13% holding an associate’s degree or completed vocational training, and 9% holding a bachelor’s or advanced degree. Participants in the current study lived in neighborhoods in a midsized city in the Southeastern United States that was characterized by high violence and/or poverty rates (e.g., neighborhoods with low-income housing and high crime rates).

### Measures

#### Demographics Questionnaire.

The Demographics Questionnaire is a parent-report measure that assesses the sex, age, and race/ethnicity of the caregiver and adolescent, current grade of the adolescent, caregiver marital status, family income, and caregiver level of education.

#### Revised Children’s Manifest Anxiety Scale (RCMAS).

The RCMAS (Reynolds & Richmond, 1985) is a 37-item self-report scale designed to measure manifest or trait anxiety in youth. Youth respond to items with a “*yes*” or “*no*” to indicate whether they feel that the statement is true for them. Higher scores indicate higher anxiety levels. The original study collected data on the 28 items that make up the total anxiety score (i.e., items comprising the lie scale were excluded). We used a modified scoring recommended by White and Farrell (2001) based on a theoretically derived structure generated by experts in child anxiety. This excludes seven items that reflect dysphoric mood and low self-concept and are believed to overlap with the construct of depression. Therefore, the final scale for the current study consisted of 21 items. Table 1 lists the original 28 items from the total anxiety scale and denotes which items were not included in the current study. The alphas for the revised total anxiety score in the current study ranged from .84 to .87 across the four waves.

#### Problem Behavior Frequency Scales–Revised (PBFS-R).

The PBFS-R is a self-report measure that consists of seven subscales that assess the frequency of problem behaviors in youth (Farrell, Kung, White, & Valois, 2000). The six-item overt and six-item relational PV subscales from the PBFS-R were used in the current study. In Wave 1, a modified version of the PBFS-R was used to determine lifetime frequency of PV. Youth were asked how many times they experienced a particular incidence of PV on a 9-point scale: 0 = *never*, 1 = *once*, 2 = *twice*, 3 = *3 or 4 times*, 4 = *5 or 6 times*, 5 = *7 or 8 times*, 6 = *at least once a month*, 7 = *at least once a week*, and 8 = *almost every day*. For Waves 2 to 4, the original rating scale of the PBFS-R was used such that respondents were asked how frequently they experience different forms of victimization (e.g., “get hit by another kid”) in the past 30 days on a 6-point scale: 0 = *never*, 1 = *1–2 times*, 2 = *3–5 times*, 3 = *6–9 times*, 4 = *10–19 times*, and 5 = *20 times or more*. The relational victimization items (e.g., “had someone spread a false rumor about you,” “been left out on purpose by other kids when it was time to do an activity”) were partially based on the Social Experiences Questionnaire (SEQ-S) developed by Crick and Grotpeter (1996). Higher scores indicate higher frequencies of PV. The PBFS-R has been used in previous evaluations of violence prevention programs with adolescents and has high internal consistency and a well-established factor structure (Farrell, Sullivan, Goncy, & Le, 2016). Alpha coefficients for these scales ranged from .78 to .85 for overt victimization and from .79 to .86 for relational victimization.

### Procedures

The study was approved by the Institutional Review Board at Virginia Commonwealth University. Based on the aims of the larger study, participants were recruited from neighborhoods within a midsized southeastern city in the United States and neighboring counties with high levels of violence and/or poverty according to police statistics and 2000 census data. The study was advertised through community agencies and events, and by canvassing qualifying neighborhoods via flyers posted door-to-door. To be eligible, both a female caregiver and her adolescent youth had to live in the target neighborhoods and the adolescent had to be enrolled in the fifth or eighth grade at the first wave. Eligible and interested families were scheduled for initial interviews, which began in January 2005, with follow-up interviews occurring annually for 3 years. Face-to-face interviews were conducted and in separate rooms for caregivers and adolescents primarily in participants’ homes. Sixty-three percent of eligible participants agreed to be in the study. Interviewers reviewed the caregiver consent and youth assent forms with the family. After the maternal caregiver provided written consent, the caregiver and youth separated for the interviews, and youth provided assent prior to continuing. A Certificate of Confidentiality was obtained from the National Institutes of Health (NIH) to protect families’ responses. The interviewers hired to conduct these sessions were of various racial/ethnic backgrounds and sexes. Tests for interviewer race and sex effects revealed no systematic biases, *p*s > .10. Interviews with the caregiver and youth lasted approximately 90 min, included assistance with self-report measures, and participants received US$50 in gift cards per family at each wave. At the end of the study, names of families who finished all four interviews were put in a drawing for US$300, US$200, and US$100 prizes. Sixty-nine percent of the original sample was retained across the entire four-wave study. These retention rates are better than many community-based studies for recruiting participants from high-risk neighborhoods (Luthar & Goldstein, 2004). The present study was completed through secondary data analyses using de-identified data from this previously IRB approved study (IRB# B-HM3768).

### Data Analysis

Descriptive statistics were calculated to examine the distribution properties of each scale and to detect any outliers. Next, attrition was examined using *t* tests on baseline data to see whether youth who completed all four waves of data differed from those who dropped out of the study. We also conducted a test to determine whether the missing data were missing completely at random (MCAR). Then, correlations between anxious symptoms, overt victimization, and relational victimization were calculated to examine the relations within each of the four waves of data using MPlus Version 6.1 (Muthén & Muthén, 2012) and full-information maximum likelihood (FIML; Schafer & Graham, 2002). Significance for all tests was established at an alpha level of .05, two-tailed.

**Figure 1.**
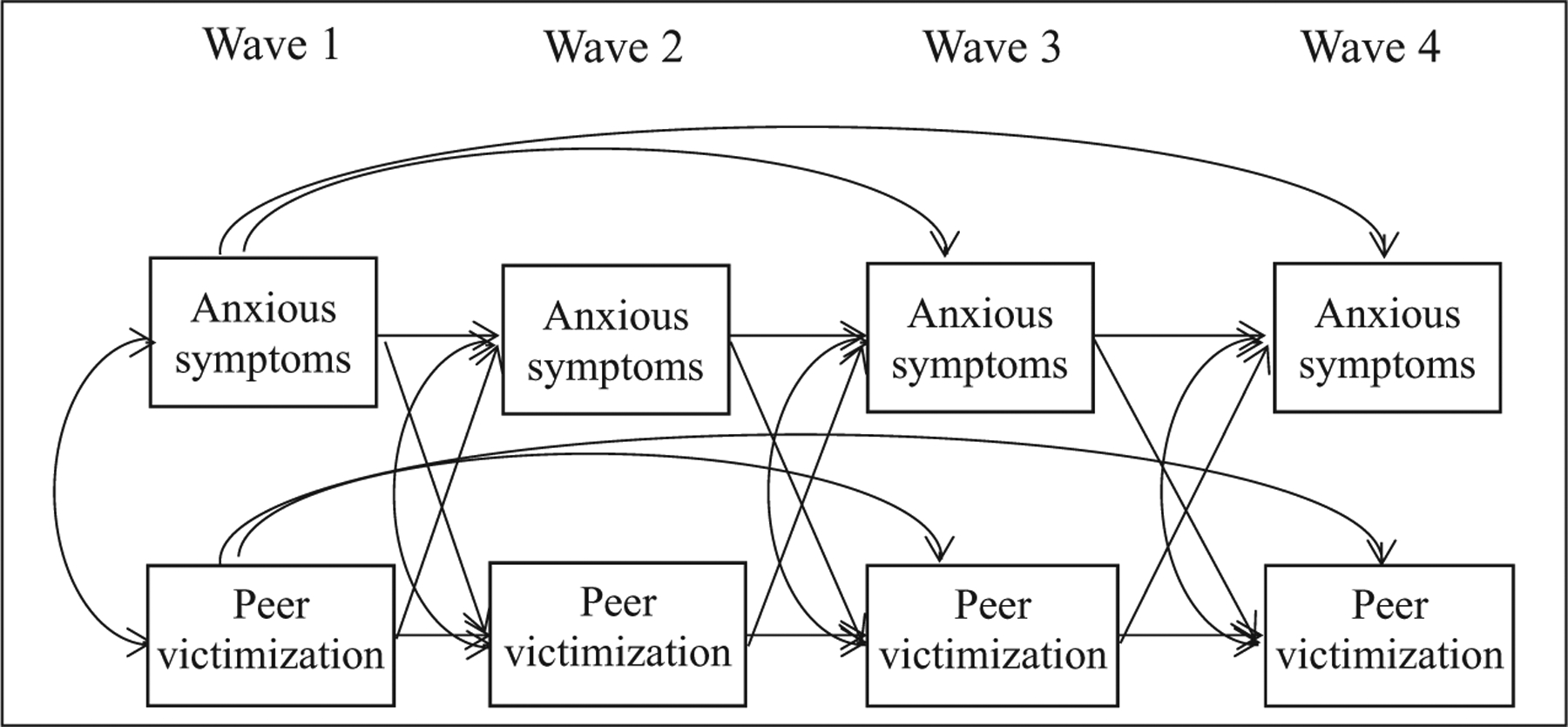
Full model examining the bidirectional relations between anxious symptoms and peer victimization (Model 4). *Note.* The same model was used for overt and relational victimization.

We used a series of path models to examine bidirectional relations between anxious symptoms and *overt victimization* and between anxious symptoms and *relational victimization*. To reduce the likelihood of committing a Type I error, each model we successively built from the least to most complex model resulting in four progressively more complex models. Then, we compared each model using the fit indices described below as well as the Satorra–Bentler scaled chi-square which takes into account the scaling correction factor for the robust maximum likelihood (MLR) estimator used to accurately conduct chi-square difference testing. The models were (a) an autoregressive model in which each variable was regressed the prior level of that variable (Model 1); (b) an anxiety prediction model in which previous waves of anxiety were added to the autoregressive model to predict future waves of victimization (Model 2); (c) a victimization prediction model in which previous waves of victimization were added to the autoregressive model to predict future waves of anxiety (Model 3); and (d) a full bidirectional model in which both previous waves of victimization and anxiety were added to the autoregressive model to predict future waves of each construct (Model 4, see Figure 1). Model 4 tested the bidirectional hypothesis.

The following criteria were used to assess a good fit for the models: (a) chi-square to degrees of freedom ratio less than 2.0; (b) the comparative fit index (CFI) of more than .95 (Hu & Bentler, 1999); and (c) the root mean square error of approximation (RMSEA) close to .06 or less (the RMSEA uses errors of prediction and measurement to assess the degree of match between the hypothesized and true models; Tabachnick & Fidell, 2001).

## Results

### Descriptive Statistics

We followed procedures recommended by Tabachnick and Fidell (2001) to address several highly skewed variables and limit the impact of extreme values by recoding scores that exceeded a *z*-score of 3.29 to a score equivalent to a *z*-score of 3.29. We used the winsorized data in all analyses. In addition, we conducted the path analysis using an estimator robust to nonnormality (i.e., MLR). Table 2 reports the sample size, means, standard deviations, and the Pearson correlations using FIML for Waves 1 through 4 for all study variables and denotes which variables were winsorized. The study variables were all significantly positively correlated with each other, with the exception of Wave 1 anxiety and Wave 4 relational PV (*r* = .10, *ns*). Our attrition analyses did not identify any significant differences between participants who completed all of the study measures at all waves and those who did not on any demographics or study variables. Furthermore, according to Little’s (1988) chi-square statistic data were MCAR, χ^2^ = 161.82, *df* = 139, *p* = .090.

### Path Analyses Models

#### Relations between anxious symptoms and overt PV.

Comparison of the four models examining relations between anxious symptoms and overt PV indicated that Model 2, in which anxiety predicted subsequent changes in overt PV, improved upon the fit of the baseline model (Model 1; see Table 3 for fit statistics). The addition of paths in which overt PV predicted subsequent changes in anxiety did not improve upon the fit of the baseline model, χ^2^Δ(3) = 4.52, *p* = .21, or of Model 2 model, χ^2^Δ(3) = 4.43, *p* = .22. Thus, our findings supported the notion that anxiety predicted subsequent changes in overt PV but did not support reciprocal relations such that overt PV predicted changes in anxiety. Standardized path coefficients indicated that anxiety predicted changes in overt PV across Waves 1 and 2 and across Waves 2 and 3, but not across Waves 3 and 4 (see Table 4). Consistent with correlational analyses, all previous wave levels of either anxious symptoms or overt PV predicted the following year’s levels of the same construct (e.g., Wave 1 anxious symptoms predicted Wave 2 anxious symptoms, Wave 2 anxious symptoms predicted Wave 3 anxious symptoms, etc.) across all models tested.

#### Relations between anxious symptoms and relational PV.

Comparison of the four models examining relations between anxious symptoms and relational PV indicated that Model 4, the bidirectional model, in which anxiety predicted subsequent changes in relational PV and relational PV predicted subsequent changes in anxiety improved upon the fit of all other models (see Table 3 for fit statistics).

Similar to overt PV, standardized path coefficients indicated that anxiety predicted changes in relational PV across Waves 1 and 2 and across Waves 2 and 3, but not across Waves 3 and 4 (see Table 4). Once more, all previous wave levels of either anxious symptoms or relational PV predicted the following year’s levels of the same construct. However, unique to the relational PV model, more relational PV at Wave 1 predicted more anxiety at Wave 2.

## Discussion

The current study examined bidirectional relations between anxious symptoms and two forms of PV (i.e., overt and relational) within a community sample of predominantly African American adolescents living in low-income urban areas across four waves of data collected annually during key transition periods (i.e., elementary to middle school; middle school to high school) and the following 2 years. We expected to find bidirectional, longitudinal relations between anxious symptoms and PV over time.

Anxious symptoms predicted both overt and relational PV, but only at the time of transition (Wave 1 to Wave 2) and the following year (Wave 2 to Wave 3). This expands on our current knowledge that has found varying levels of support for the notion that anxiety “drives” risk for PV. This extends findings from studies that showed social phobia symptoms predicted overt PV in certain populations (e.g., Finnish adolescent boys, Ranta et al., 2012) and relational PV in others (e.g., majority Hispanic adolescents, Siegel et al., 2009; adolescents at an urban parochial high school, Storch, Masia, Warner, Crisp, & Klein, 2005). However, these findings were contrary to previous work that found that social anxiety symptoms did not predict overt PV (Siegel et al., 2009). The differences in results may be explained by the different populations researched, the limited time period investigated in previous work, and the focus on social anxiety as compared with a more broad set of anxious symptoms. Furthermore, a more generalized view of anxiety symptoms may be better at explaining future PV experiences, especially during times of transitions, as compared with focusing solely on social anxiety symptoms.

In addition, although previous levels of PV and future anxious symptoms were positively correlated over time, only relational PV at Wave 1 predicted anxious symptoms at Wave 2. This mirrors findings by others that relational PV, but not overt PV, predicted social anxiety symptoms (Siegel et al., 2009). These results highlight that when youth transition between school settings, they may be more vulnerable to previous experiences of relational PV. Furthermore, anxious symptoms maybe more important in explaining future PV compared with PV explaining future anxiety symptoms. Indeed, other work investigating depressive symptoms has found that internalizing symptoms predict PV but not vice versa (Kochel et al., 2012; Tran et al., 2012). This study provides evidence that anxious symptoms work in a similar fashion.

These findings add limited support for previously discussed theories and research that suggest a bidirectional relation between anxious symptoms and PV (e.g., Bernstein & Watson, 1997; Storch & Ledley, 2005), and internalizing symptoms more broadly (e.g., Boivin, Petitclerc, Feng, & Barker, 2010). The present study’s findings highlight the importance of investigating the precise type of PV, as well as exploring the relations over an extended time period as suggested by previous researchers (Reijntjes et al., 2010). In addition, youth’s environment and culture, based on a variety of factors, but not limited to country of origin, socioeconomic status, and sex, may play an important and significant role in how anxiety and PV relate over time.

Also, although not a main focus of the study, it is important to note that previous levels of each construct (i.e., anxious symptoms, PV) consistently predicted the following year’s levels of the construct across all models tested. Researchers who have included analogous paths in their investigations also have found that previous levels of internalizing symptoms and PV significantly and consistently predicted future levels of each construct, respectively (Boivin et al., 2010; Kochel et al., 2012; Tran et al., 2012). Therefore, as expected, the most effective interventions or prevention methods for a particular problem should focus on that concern. However, if resources are limited focusing on anxiety symptoms may be beneficial. This may be especially important as in the current study, anxiety symptoms appeared more robust over time as Wave 1 anxious symptoms continued to predict future anxiety symptoms at Wave 4. This pattern was not observed with either type of PV.

Given this study’s findings, it is important to consider what current PV programs tend to offer to youth and if this is sufficient for reducing instances of PV. Programs that focus on reducing PV, as well as bullying, typically reduce PV by 17% to 20% on average (Ttofi & Farrington, 2011). According to parents, teachers, and self-reports, youth with anxiety disorders are rated as less socially competent than other youth (e.g., Chansky & Kendall, 1997), lack supportive positive interactions with friends and classmates (La Greca & Lopez, 1998), and avoid social interactions with peers (Gazelle & Rudolph, 2004). These factors may put these youth at risk for PV. In addition, because youth with anxiety are fearful and their thoughts focus on anticipated harm or danger they may be more responsive to PV (e.g., get visibly afraid or upset), as compared with their nonanxious peers (Hodges & Perry, 1999). All of these topics should be addressed in PV programs.

However, although most programs used in the United States include assertiveness and social skills training (e.g., Olweus Bullying Prevention Program [OBPP], Melton et al., 1998; Steps to Respect, Frey et al., 2005; Youth Matter, Jenson & Dieterich, 2007), few program directly address anxious symptoms although there are some exceptions (e.g., Social Skills Group Intervention [S.S.GRIN], DeRosier & Marcus, 2005; School Psychiatric Consultation [SPC], Fonagy et al., 2009). By neglecting to focus on the anxious symptoms which put youth at greater risk for PV relative to nonanxious peers, particularly during times of transition, the current programs appear to focus more on intervening on the perpetrator side of the situation as compared with the victim side. Given that the majority of youth involved in PV report both being a perpetrator and a victim, commonly called “bully-victims” (e.g., Nansel et al., 2001; Schwartz, 2000), creators and schools that implement these programs should consider adding components that target coping with anxious symptoms. Such interventions should be created from evidence-based treatments which have been found to be effective in reducing anxiety symptoms in this population (e.g., cognitive behavioral therapy; Higa-McMillan, Francis, Rith-Najarian, & Chorpita, 2016).

### Study Limitations and Directions for Future Research

Although the study had many methodological strengths, it is important to note the limitations that may have impacted the results. First, all of the data were adolescents’ self-reports of anxious symptoms and PV, which raises concerns of shared method variance and socially desirable responding (Kazdin, 2003). Using more than one reporter for variables would have made it possible to examine the correspondence of findings based on the reporting source, or to combine measures of the same construct into a robust measure of anxiety or PV, which could have reduced these concerns. However, several studies have demonstrated the reliability of self-report measures of PV (e.g., Crick & Bigbee, 1998; Crick & Grotpeter, 1996; Prinstein, Boergers, & Vernberg, 2001), and the scales used in the current study were based on measures that are comparable to peer-nomination measures of PV (Crick & Bigbee, 1998). In addition, other research has found support that adolescents are more accurate reporters of their internalizing symptoms than other reporters (e.g., Holmbeck, Li, Schurman, Friedman, & Coakley, 2002). Therefore, youth report may be appropriate to use in the present study’s context.

Second, sex and age were not examined as moderators. Because parameters double when unconstrained multiple group analyses are conducted (e.g., estimating parameters separately for boys and girls; Kline, 1998), it was determined that the present study would likely be unpowered to conduct these types of analyses. Therefore, given the concerns about statistical power, no moderation analyses were conducted. However, examination of the zero-order correlations across sex and age revealed few differences. Ideally, a study with a large enough sample size would be able to create an even more comprehensive model that includes both types of PV and various internalizing symptoms that can then be tested for moderation by age and sex through multiple group analyses, while also controlling for other demographics.

Third, this study considered anxious symptoms broadly. However, anxiety presents in a variety of ways in youth, and these may lead to differential diagnoses (e.g., generalized anxiety, social phobia, separation anxiety, panic; American Psychiatric Association, 2013). Past work which has focused on a specific presentation primarily has investigated social phobia (e.g., Ranta et al., 2012; Siegel et al., 2009) because one of the theorized goals of PV is to affect a youth’s social network (Crick, Grotpeter, & Bigbee, 2002). Future studies should investigate how different presentations of anxiety in youth may explain varying levels of PV over time, particularly in samples not yet investigated (e.g., low-income urban youth).

Fourth, the timing of this study’s assessments needs to be taken into consideration. All constructs were assessed 1 year apart. This time period may be too long to observe changes, as the relations may have a more immediate effect as observed in studies with shorter time frames (e.g., Sinclair et al., 2012). Upon closer examination of the correlations, youth’s reports of anxious symptoms were much more stable across time than their reports of PV. Given that youth’s social relationships and identity development are frequently changing during the developmental stages investigated (i.e., middle and late adolescence, Lerner & Steinberg, 2004), which included the transition to middle school or high school, a 1-year time gap may not accurately assess the changes occurring between these constructs especially considering youth reported on their past 30-day experiences with PV and overall trait anxiety. Given the instability of PV across time, it can by hypothesized that this construct is more varied, and how close the assessment was to an incident of PV may have influenced the results. However, there is evidence that self-reported PV is moderately stable across a 1-year interval (Pouwels, Souren, Lansu, & Cillessen, 2016). Nevertheless, future studies should consider more frequent assessment time points to gain a better understanding of how the timing of PV affects a youth’s feelings of anxiety.

Furthermore, more research is needed to assess the generalizability of the current study’s findings to adolescents from different contexts (e.g., rural, suburban), ethnic/racial backgrounds, or socioeconomic status (SES) levels, as the sample for this study was predominately African American urban adolescents from low-income families. In addition, most of the data collected in this study occurred before there was widespread usage of smartphones and social media by youth, which has changed the patterns of adolescent communication and the PV landscape to include more instances of PV by indirect means, especially through cyberbullying (Kowalski, Giumetti, Schroeder, & Lattanner, 2014).

### Summary

This study contributed to our understanding of the prospective relations between anxiety symptoms and overt and relational PV in a commonly underrepresented population in the current research. Similar to past research which has included analogous paths in their investigations (Boivin et al., 2010; Kochel et al., 2012; Tran et al., 2012), this study found that previous levels of anxious symptoms and PV significantly and consistently predict future levels of each construct, respectively. Consequently, intervention and prevention programs which want to reduce a specific problem should start by targeting the symptom/behavior of interest. In addition, anxious symptoms during times of transition and up to 1 year following the transition predicted both overt and relational PV experiences, while only relational PV during the transition year predicted anxious symptoms. Therefore, developing and testing interventions that target anxious thoughts and feelings during these key times in adolescence should be a priority.

## Figures and Tables

**Table 1.** Revised Children’s Manifest Anxiety Scale (RCMAS) Original Study Items and Items Excluded for Current Study.

Item	
1.	I have trouble making up my mind.
2.	I get nervous when things do not go the right way.
3.	*Others seem to do things easier than I can.*
4.	Often I have trouble getting my breath.
5.	I worry a lot of the time.
6.	I am afraid of a lot of things.
7.	*I get mad easily.*
8.	I worry about what my parents will say to me.
9.	I feel like others do not like the way I do things.
10.	It is hard for me to sleep at night.
11.	I worry about what other people think of me.
12.	*I feel alone even when there are people with me.*
13.	Often I feel sick to my stomach.
14.	My feelings get hurt easily.
15.	My hands feel sweaty.
16.	*I am tired a lot.*
17.	I worry about what is going to happen to me.
18.	*Other children are happier than I am.*
19.	I have bad dreams.
20.	My feelings get hurt easily when I am fussed at.
21.	I fear someone will tell me I do things the wrong way.
22.	I wake up scared some of the time.
23.	I worry when I go to bed at night.
24.	*It is hard for me to keep my mind on my schoolwork.*
25.	I wiggle in my seat a lot.
26.	I am nervous.
27.	*A lot of people are against me.*
28.	I often worry about something bad happening to me.

*Note*. Italics denote item was excluded in the current study.

**Table 2.** Means, Standard Deviations, Sample Sizes, and Correlations Between Study Variables.

Variable		*n*	*M* (SD)	1	2	3	4	5	6	7	8	9	10	11	12
1.	Anxiety—Wave 1	355	7.40 (4.86)	—											
2.	Anxiety—Wave 2	318	5.48 (4.72)	.59***	—										
3.	Anxiety—Wave 3	270	5.02 (4.49)	.53***	.68***	—									
4.	Anxiety—Wave 4^a^	245	5.08 (4.23)	.45***	.53***	.70***	—								
S.	Overt Victimization—Wave 1^a^	358	8.31 (7.88)	.35***	.23***	.13*	.13*	—							
6.	Overt Victimization—Wave 2^a^	317	3.35 (4.17)	.32***	.43***	.29***	.17*	.45***	—						
7.	Overt Victimization—Wave 3^a^	271	2.84 (3.71)	.20**	.32***	.31***	.13*	.31***	.46**	—					
8.	Overt Victimization—Wave 4^a^	247	2.40 (3.44)	.13*	.26***	.23**	.22***	.18**	.36***	.49***	—				
9.	Relational Victimization—Wave l^a^	358	6.96 (7.27)	.43***	.37***	.22***	.22***	.62***	.41***	.27***	.18*	—			
10.	Relational Victimization—Wave 2^a^	317	3.02 (3.95)	.35***	.51***	.31***	.25***	.37***	.68***	.37***	.25***	.49**	—		
11.	Relational Victimization—Wave 3^a^	271	2.55 (3.66)	.26***	.42**	.46***	.29***	.17**	.35***	.66***	.33***	.32**	.48**	—	
12.	Relational Victimization—Wave 4^a^	247	2.43 (3.52)	.10	.26***	.25***	.38***	.14*	.17*	.28***	.64***	.24**	.21**	.35***	—

a Variable winsorized to account for nonnormality.

* *p* < .05.

** *p* < .01.

*** *p* < .001.

**Table 3.** Path Model fit Statistics for the Bidirectional Relations Between Anxious Symptoms and Peer Victimization.

		χ^2^	*df*	CFI	RMSEA	Satorra-Bentler Scaled χ^2^	*P*	Comparison Model
Relations between anxious symptoms and overt peer victimization								
1.	Autoregressive model	27.11**	14	.97	.05			
2.	Anxiety predicting overt victimization	11.20	11	1.00	.01	18.02	<.001	1
3.	Overt victimization predicting anxiety	22.51*	11	.98	.05	4.52	.210	1
4.	Full model	6.92	8	1.00	.00	21.74	.001	1
						4.43	.219	2
						17.91	<.001	3
Relations between anxious symptoms and relational peer victimization								
1.	Autoregressive model	35.61**	14	.96	.07			
2.	Anxiety predicting relational victimization	14.26***	11	.99	.03	22.49	<.001	1
3.	Relational victimization predicting anxiety	25.16**	11	.98	.06	10.51	.015	1
4.	Full model	5.60	8	1.00	.00	31.03	<.001	1
						8.87	.031	2
						20.79	<.001	3

Note. CFI = comparative fit index; RMSEA = root mean square error of approximation.

* *p* < .05.

** *p* < .01.

*** *p* < .001.

**Table 4.** Standardized Estimates of Best Fitting Models for Overt and Relational Victimization.

Path	Beta	*P*
Model 2. Anxiety predicting overt victimization	.59	< .001
Anxiety W1 → Anxiety W2		
Anxiety W1 → Anxiety W3	.98	.001
Anxiety W2 → Anxiety W3	.57	< .001
Anxiety W1 → Anxiety W4	.12	.044
Anxiety W3 → Anxiety W4	.64	< .001
Victimization W1 → Victimization W2	.38	< .001
Victimization W1 → Victimization W3	.13	.093
Victimization W2 → Victimization W3	.35	< .001
Victimization W1 → Victimization W4	.02	.8l5
Victimization W3 → Victimization W4	.47	< .001
Anxiety W1 → Victimization W2	.20	< .001
Anxiety W2 → Victimization W3	.14	.043
Anxiety W3 → Victimization W4	.08	.235
Model 4. Bidirectional relations between relational victimization and anxiety		
Anxiety W1 → Anxiety W2	.52	< .001
Anxiety W1 → Anxiety W3	.21	< .001
Anxiety W2 → Anxiety W3	.59	< .001
Anxiety W1 → Anxiety W4	.14	.017
Anxiety W3 → Anxiety W4	.65	< .001
Victimization W1 → Victimization W2	.42	< .001
Victimization W1 → Victimization W3	.10	.096
Victimization W2 → Victimization W3	.32	< .001
Victimization W1 → Victimization W4	.12	.106
Victimization W3 → Victimization W4	.26	.001
Anxiety W1 → Victimization W2	.17	.005
Anxiety W2 → Victimization W3	.22	.006
Anxiety W3 → Victimization W4	.11	.115
Victimization W1 → Anxiety W2	.15	.007
Victimization W2 → Anxiety W3	−.06	.338
Victimization W3 → Anxiety W4	−.05	.424
